# Evaluation of the effects of a calcineurin inhibitor in experimental models of Alzheimer’s disease

**DOI:** 10.1002/alz.086388

**Published:** 2025-01-03

**Authors:** Sarah Tavares Araújo Santos, Giovanni Freitas Gomes, Nádia Gomes Rocha, Antônio Carlos Pinheiro Oliveira

**Affiliations:** ^1^ Universidade Federal de Minas Gerais, BELO HORIZONTE, Minas Gerais Brazil; ^2^ Universidade Federal de Minas Gerais, Belo Horizonte Brazil; ^3^ Universidade Federal de Minas Gerais, Belo Horizonte, Minas Gerais Brazil

## Abstract

**Background:**

Calcineurin, a protein involved in functions such as synaptic plasticity and neuronal survival, plays an important role in the pathophysiology of Alzheimer’s disease. This study, randomized, investigated the effects of FK506 (FK), a calcineurin inhibitor, on the behavioral, histological, and biochemical alterations observed in models of neurotoxicity induced by NMDA or Aβ and in a transgenic model for AD, in addition to the organotypic culture model stimulated with NMDA.

**Methods:**

This study involved models for AD to experiment injecting NMDA or Aβ1‐42 into the hippocampus of male C57Bl/6 mice aged 8‐12 weeks to induce a neurotoxicity model, treating double‐transgenic APP/PS1 mice, expressing both mouse/human APP and mutant human PS1, with chronic FK506 for AD, in which, to enable NMDA or Aβ1‐42 microinjections, another experiment including a stereotaxic surgery was performed on the C57Bl/6 mice. FK506 doses and the pre‐treatment time were chosen based on prior related studies and its ability to cross the BBB. To obtain organotypic cultures, animals aged between 1 and 3 days were used. Their hippocampi were dissected in an ice‐cold culture and then stimulated with NMDA, which is neurotoxic, or PBS, having previously been inserted into a medium containing FK506 or a negative control. In addition, the microglia depletor clodronate disodium was added to the hippocampal slices. The exploratory open field task, the elevated plus maze (LCE), the interaction test (IS) and social memory and ORT were used to evaluate behavior. To complement the results, real‐time quantitative PCR and a statistical analysis of the data were carried out.

**Results:**

FK506 played a protective role in cognitive impairment in both *in vivo* models and reduced FJC labeling and microgliosis in the Aβ model. FK506 also prevented the reduction of NeuN, a mature neuron marker, expression in organotypic cultures stimulated with NMDA. Furthermore, chronic treatment with FK506 protected against the behavioral alterations observed in LCE and IS. In addition, FK506 reduced microgliosis in the hippocampus of APP/PS1 animals.

**Conclusion:**

Together, these results demonstrate the protective potential of FK506 against the pathology observed in AD models.

